# Nanosomal-Mediated Lipid Suspension Delivery of Docetaxel as a Promising Landscape to Enhance the Therapeutic Potential in Triple-Negative Breast Cancer

**DOI:** 10.2174/0118715206366378250519105734

**Published:** 2025-09-18

**Authors:** Pritya Jha, Varisha Anjum, Rabia Choudhary, Ammar Kadi, Faraat Ali, Irina Potoroko

**Affiliations:** 1 Department of Pharmacy, Banasthali University, Rajasthan, India;; 2 Department of Food Technology and Biotechnology, South Ural State University, Chelyabinsk, Russia;; 3 Sunder Deep Pharmacy College, Sunder Deep Group of Institutions, Ghaziabad, Delhi-Hapur Expressway, Dasna, Uttar Pradesh, India;; 4 Department of Analytical Chemistry, Faculty of Pharmacy in Hradec Králové, Charles University, Heyrovského 1203/8, 500 03, Hradec Králové, Czech Republic

**Keywords:** Breast cancer, HER2 expression, breast cancer metastasis, triple-negative breast cancer, docetaxel, systemic toxicity, taxanes

## Abstract

The challenging subtype of breast cancer known as Triple-Negative Breast Cancer (TNBC) is characterized by the absence of HER2 expression, progesterone receptors, and estrogen receptors. TNBC is linked to a harsh treatment trajectory, elevated rates of recurrence, and restricted therapeutic alternatives. The mainstay of treatment for TNBC has historically been conventional chemotherapy, especially taxanes like Docetaxel. However, the effectiveness of these drugs is frequently compromised by systemic toxicity and resistance mechanisms. The development of Nanosomal Docetaxel Lipid Suspension (NDLS) offers a promising alternative, designed to enhance Docetaxel's therapeutic index by improving solubility, reducing side effects, and optimizing tumor-targeted drug delivery. NDLS has potential as a delivery system for additional chemotherapy drugs or combination treatments. This study addresses the cellular and molecular causes of TNBC, emphasizes the drawbacks of traditional treatments, and offers a thorough examination of NDLS in preclinical and clinical settings. This review provides a thorough analysis of NDLS in TNBC, laying the groundwork for further studies and therapeutic applications.

## 1 INTRODUCTION

Genetic alterations in germinal or somatic cells, including inherited or irregular ones, can lead to malignancies, such as breast cancer, which is a prevalent global disease [1]. Breast Cancer (BC) is the most widespread cancer among women, accounting for 30% of cases. TNBC, a subtype, accounts for 10%-15% of identified cases, presenting a significant unmet need [2]. TNBC is characterized by the absence of progesterone and estrogen receptors, along with a lack of overexpression of the human epidermal growth factor receptor 2 (HER2) [3-5]. TNBC is a biologically and clinically diverse disease that is more frequent in younger women and women with a BRCA1 gene mutation. Chemotherapy was the only systemic treatment available for this subtype until a few years ago [2].

Tumor Microenvironment (TME), specific to TNBC, is distinct from those of other subtypes. TME is linked to immune system suppression, angiogenesis, promotion of proliferation, inhibition of apoptosis, and treatment resistance [6]. Triple Negative Breast Cancer (TNBC) is an uncommon cancer that frequently leads to distant metastases, particularly in the central nervous system and lungs. Treatment involves cytotoxic chemotherapy, including anthracyclines, platinum derivatives, and taxanes, and may include radiation and surgery. However, only brief PFS and OS have been reported [7]. Taxanes docetaxel and paclitaxel, licensed in Europe 10 years ago, have become standard treatments for breast cancer and are well-known anti-mitotic chemotherapy medications used as the first line of treatment for various cancers [8]. Research is underway to determine their potential role in adjuvant therapy for early-stage disease, as clinically significant advantages were initially demonstrated in metastatic situations and neoadjuvant settings, primarily with docetaxel [9-11].

For the past 20 years, Docetaxel has been a commonly used second-generation taxane. It is made by semisynthesis, starting with a precursor that is taken from the yew plant's renewable needle biomass [12]. It is nearly insoluble in water and extremely lipophilic. The (FDA) Food and Drug Administration has approved insoluble docetaxel, commonly known as Taxotere, for the treatment of various cancers, including locally advanced or metastatic breast cancer, non-small cell lung cancer, and ovarian cancer. It can also be used in combination with other medications to treat multiple different cancer types, including gastric adenocarcinoma, prostate cancer, and head and neck cancer [13]. The conventional formulation of docetaxel presents toxicity concerns due to the presence of ethanol and polysorbate 80 excipients, leading to side effects such as alcohol intoxication, anaphylactoid reactions, peripheral neuropathy, fluid retention, and acute hypersensitivity responses. Despite premedication with corticosteroids and antihistamines, some individuals still experience severe side effects [14, 15].

The Nanosomal Docetaxel Lipid Suspension (NDLS) was formulated using lipids that are recognized as safe (GRAS) by the FDA, excluding the inclusion of ethanol or polysorbate 80 [16]. In a prospective study, NDLS was found to be as effective and tolerable as traditional docetaxel in treating metastatic breast cancer. Moreover, NDLS has been confirmed to be safe and effective in the treatment of breast cancer in earlier retrospective investigations [17]. This paper reviews the use of NDLS-based chemotherapy in breast cancer treatment, with potential applications for triple-negative breast cancer.

## 2 METHODOLOGY

The role of Nanosomal Docetaxel Lipid Suspension (NDLS) in Triple-Negative Breast Cancer (TNBC) was examined in this review using a methodical way. Several internet databases, including PubMed, Scopus, Web of Science, and Google Scholar, were searched extensively for relevant material using terms such as “triple-negative breast cancer,” “docetaxel,” “nanosomal lipid suspension,” and “NDLS chemotherapy.”

Preclinical, clinical, and mechanistic studies published between 2000 and 2024 were among the studies considered in this analysis. Only retrospective observational studies, clinical trials (phases I–III), and peer-reviewed publications that addressed NDLS in TNBC were taken into account. Studies evaluating NDLS pharmacokinetics, therapeutic effectiveness, safety profile, and mechanisms of action in comparison to traditional docetaxel formulations were required to meet the inclusion criteria. In order to compare NDLS with other cutting-edge therapies, articles addressing alternative nanotechnology-based drug delivery methods were also assessed.

Drug formulation, patient demographics, trial design, treatment outcomes (*e.g.*, progression-free survival, overall survival, response rates), and side effects were among the important factors that were the focus of data extraction. Using standards like sample size, statistical analysis, and potential biases, studies were evaluated critically for methodological quality. To evaluate the effect of NDLS on TNBC management, meta-analytical insights were extracted from pooled data where appropriate. The goal of this review is to present a thorough assessment of NDLS's therapeutic potential, benefits over traditional chemotherapy, and prospective applications in clinical cancer in the future.

## 3 MOLECULAR AND CELLULAR BASIS OF BREAST CANCER

Breast cancer is the most Common worldwide cancer, with 310,720 new cases in the US in 2024, including invasive and non-invasive cases, in addition to skin cancers [18]. Breast cancer risk factors include genetics, acquired environmental factors, and environmental factors that have been reported. Health issues can be influenced by various factors such as toxins, obesity, age, pollution, elevated estrogen, smoking, and alcohol consumption. This includes factors like early menarche, late menopause, delayed childbirth, hormone replacement therapy, and certain occupational exposures [19].

From a molecular standpoint, the most significant molecular targets linked to breast cancer are epidermal growth factor 2 and estrogen receptor α, a transcription factor that may activate neoplastic growth pathways [20]. Age is the most significant risk factor for breast cancer, with a positive family history being the next most influential factor. Women who inherit loss-of-function mutations have a 70% likelihood of developing invasive breast cancer by the age of 70 [21]. The two main factors related to an increased likelihood of breast cancer are BRCA1 and BRCA2 mutations. Germ-line mutations in BRCA 1 and 2 are responsible for sixteen percent of all hereditary breast cancers [22]. Triple-negative breast cancer, the most aggressive subtype of breast cancer for which treatment is medically challenging, can originate from another extremely significant mutation in the TP53 gene [23]. Numerous Cancer Predisposition Genes (CPGs) associated with Breast Cancer (BC) have been identified. The results of two large-scale case-control studies analyzing the correlation between BC risk and germline pathogenic variants in CPGs related to BC were recently published. Dorling *et al.* examined a panel of 34 known or suspected Breast Cancer (BC)-associated Cancer Predisposition Genes (CPGs) in 60,466 women with BC and 53,461 controls [24]. Another study [25] investigated a panel of 28 known or suspected breast cancer-associated CpG sites in 32,247 women with BC and 32,544 controls.

Notably, the majority of these publications have both relied solely on the age at diagnosis and neglected to consider additional phenotypic alterations, like tumor stage and tumor receptors, or have been based on relatively small cohorts [26]. Small molecule medicines have the potential to target dysregulated epigenetic pathways, in contrast to genetic mutations that are difficult to rectify. Moreover, epigenome modification across a spectrum of solid tumors makes cancer cells more vulnerable to immune system assaults and increases their susceptibility to immunotherapy [27, 28]. Epigenetics is a heritable biological mechanism that modifies the expression of genes without changing the DNA sequence itself, under the direction of outside stimuli [29]. Epigenetics-based diagnostic and prognostic approaches hold considerable promise for precision oncology, with several DNA methylation diagnostic tests currently in clinical trials or already in use. Furthermore, efforts in precision oncology to address dysregulated epigenetic pathways have led to the development of epidrugs [30].

Another result of efforts in precision oncology to address dysregulated epigenetic pathways is the development of epigenetic drugs, which target epigenetic modulators. Currently, the FDA has approved only nine epidrugs, including inhibitors of EZH2, IDH, Histone Deacetylases (HDACis), and DNA Methyltransferases (DNMTs). The others are still in clinical testing. Table **1** represents the FDA-approved epigenetic therapy [31-37].

## 4 BREAST CANCER METASTASIS

Cancer metastasis, the advancement of secondary malignancies in distant organs, is the primary cause of mortality for most breast cancer patients, and breast cancer cells often spread to other organs, facing environmental obstacles and tissue-resident cells that create barriers preventing tumor growth and survival [38]. Breast cancer metastases cause over 90% of cancer-related deaths, with up to 60%-75% of patients experiencing bone, 32-27% lung, 32-25% liver, and 10% other metastases, according to recent studies [39, 40].

Metastasis refers to the complex processes through which a primary tumor spreads to distant secondary sites, often contributing to treatment failure and a high number of cancer-related deaths [40]. It plays a pivotal role in a patient's prognosis, as it represents the advanced stage of most cancers and involves several cellular mechanisms such as invasion, immune evasion, tumor proliferation, and modification of the tissue microenvironment [41]. In breast cancer, metastasis is a key therapeutic target and a significant indicator for treatment decisions. Targeted therapies aim to block molecules vital for tumor cell survival and growth, influenced by factors such as hormone receptor status, HER2 overexpression, metastasis rate, and the location of metastasis [41-43]. Fig. (**1**) illustrates the metastasis pattern in TNBC. Personalized treatment strategies can reduce chemotherapy-associated side effects and improve outcomes for breast cancer patients. However, metastasis and recurrence remain major challenges, and no established treatment exists for Triple-Negative Breast Cancer (TNBC), which often metastasizes to the brain, bones, liver, and lungs [44]. Currently, there is no standard chemotherapy protocol for TNBC patients who have undergone prior treatment, largely due to insufficient research on molecular subtypes. Treatment strategies generally involve the use of approved drugs for breast cancer patients. Standard chemotherapy regimens, such as taxanes and anthracyclines, offer limited options for patients who relapse and later have a disease-free interval of 6-12 months. Patients are recommended to begin rechallenge regimens involving anthracyclines and taxanes. However, there is limited research on the effectiveness of these regimens as first- or second-line treatments for metastatic breast cancer [45]. To date, only one prospective phase III trial has investigated anthracycline rechallenge (NCT00091442).

## 5 TRIPLE-NEGATIVE BREAST CANCER (TNBC)

BC is a complex condition and has various histologic types, natural history, clinical behaviour, and treatment response. Over the past 15 years, breast tumors have been classified based on ER, PR, and HER2 expression. In the 2000s, anti-HER2 medications showed efficacy, and hormone receptor-expressing medicines were developed [2, 42]. Triple-Negative Breast Cancer (TNBC) is the term that is used to describe a subset of breast cancers that do not have ER, PR, or HER2. It was first used in the middle of the 2000s [43]. Triple-Negative Breast Cancer (TNBC) accounts for approximately 15% of all invasive breast cancers (BC). TNBC is more common in premenopausal women, African American women, and individuals with BRCA1 or BRCA2 mutations.It expresses HER2 and lacks estrogen/progesterone receptors, with a worse prognosis and more aggressive clinical course. Advanced diagnosis, earlier metastasis recurrence, and lower overall survival rate are associated with TNBC [44, 45].

The only BC molecular subtype that does not overexpress the HER2 protein and does not have progesterone or estrogen receptors is TNBC. The subtypes of TNBC are varied and include Mesenchymal (M), Mesenchymal Stem-Like (MSL), Immunomodulatory (IM), Luminal Androgen Receptor (LAR), Basal-Like-1 (BL1), Basal-Like-2 (BL2), and Unstable (UNS) subtypes [46, 47]. BRCA1 and BRCA2 mutants that represent BL-subtypes, are distinguished by excessive proliferation and genomic instability together with abnormalities in cell-cycle checkpoints. Platinum-based regimens affect these subtypes [48]. The MSL subtype of cancer is defined by its tendency to undergo EMT and by the presence of stem cells that are responsive to drugs or treatments that inhibit EMT. This characteristic can influence how the cancer behaves and how it might be treated [49]. The IM subtype is influenced by immune signaling, while the M group of metaplastic TNBCs, which have PIK3CA mutations, can potentially be treated with PI3K inhibitors. Additionally, four TNBC molecular subtypes have been identified and reinterpreted, including Luminal Androgen Receptor (LAR), Mesenchymal, Basal-Like Immune-Suppressed (BLIS), and Basal-Like Immune-Activated (BLIA) [50]. Disease-Free Survival (DFS) for the BLIS cluster was the lowest, indicating the critical role the immune system plays in TNBC. Finding the various TNBC subtypes and molecular markers is essential to prevent designing treatment regimens in a blind manner since these characteristics are directly related to clinical outcomes, such as prognosis, responsiveness to chemotherapy, and recurrence pattern. TNBCs were studied as a diverse group of cancers using various techniques, including analysis of somatic DNA mutations, copy number changes, gene expression profiles, and immunological metagene data [51].

Small-molecule inhibitors have shown promise in treating TNBC, but drug resistance is rapidly emerging. Urgent demands include identifying TNBC molecular characteristics, focusing on tumor environment changes, and developing new treatments. TNBC's aggressiveness, medication resistance, and heterogeneity necessitate targeted therapeutic techniques and regimen combinations to improve patient outcomes [52]. Microtubule inhibition is a potential treatment for mitosis, with agents like taxane, eribulin, and vincristine being used in combination with immunotherapies or other chemotherapeutic drugs. Eribulin, a nontaxane microtubule depolymerizing agent, inhibits proliferation by binding to tubulin and microtubules. When combined with the mTOR inhibitor everolimus, these drugs significantly reduce tumor development, providing a basis for treating resistant TNBC [53].

## 6 VEGF IN BREAST CANCER: IMPLICATIONS FOR TNBC TREATMENT

Vascular Endothelial Growth Factor (VEGF) is a key regulator of angiogenesis, playing a crucial role in tumor progression and drug resistance in breast cancer, particularly In Triple-Negative Breast Cancer (TNBC). Studies have demonstrated that VEGF levels in plasma, serum, and tumor tissue correlate with tumor aggressiveness and response to therapy [54]. The pro-angiogenic environment in TNBC promotes extensive vascularization, contributing to tumor survival and resistance to chemotherapy. The expression of VEGF varies across different biological samples, with plasma, serum, and tumor tissue VEGF levels showing significant correlations with microvessel density and tumor stage. Previous research by Tae *et al*. [55] provided a comparative analysis, revealing that VEGF concentrations in tumor tissue are often higher than in circulation, indicating its role in local tumor vascularization.

Additionally, increased VEGF levels in serum and plasma have been associated with poor prognosis and therapy resistance. Although tamoxifen is primarily used for Estrogen Receptor-positive (ER+) breast cancer, it has also been shown to modulate VEGF expression. Studies suggest that tamoxifen reduces VEGF levels, impacting tumor microvasculature and potentially limiting angiogenesis-driven tumor growth [56]. This effect highlights tamoxifen’s broader implications beyond ER+ cancers and raises the possibility of exploring anti-angiogenic strategies in TNBC. Given VEGF’s critical role in TNBC progression, anti-angiogenic agents, such as bevacizumab, have been developed to inhibit VEGF signalling, thereby normalizing tumor vasculature and improving drug delivery [57]. Combining VEGF inhibitors with Nan Dispersed Lipid Systems (NDLS) offers a promising approach to enhancing drug penetration and overcoming resistance mechanisms in TNBC. Ongoing clinical trials are investigating the efficacy of combining NDLS with anti-angiogenic agents, potentially leading to innovative therapeutic strategies for aggressive breast cancers.

## 7 TAXANES

For TNBC, cytotoxic chemotherapy is presently the standard of care. First medications to be given include anthracyclines and taxanes, followed by alkylating agents, antimetabolites, and vinca alkaloids. High-grade chemosensitivity, considerable recurrence rates, and an unfavorable prognosis are characteristics of TNBC. However, the creation of novel therapeutic drugs is necessary due to the comparatively short disease-free interval and the significant adverse effects of chemotherapy regimens [58].

Taxanes are among the most important cytotoxic agents used in the treatment of various cancers. Clinical trials for the initial taxane, paclitaxel, demonstrated effective response rates and improved overall survival in patients with gastric, ovarian, breast, and lung cancers. However, paclitaxel has shown lower efficacy compared to docetaxel in certain cancers, such as advanced pancreatic tumors [59].

When paclitaxel and platinum were combined with docetaxel to treat metastatic and recurrent head and neck cancer, the response was poorer. As a result, in certain cancer types, patients receiving paclitaxel treatment may have a lower response rate than those receiving docetaxel [60].

For the past 20 years, Docetaxel, often known as Taxotere, has been a commonly used second-generation taxane. It is made by semisynthesis, starting with a precursor that is taken from the yew plant's renewable needle biomass. It is nearly insoluble in water and extremely lipophilic due to its insolubility. Docetaxel is currently formulated with polysorbate 80 and ethanol. It is FDA-approved for treating various cancers, including ovarian cancer, non-small-cell lung cancer, and locally advanced metastatic breast cancer. Additionally, it is also authorized for use in combination with other drugs to treat different cancer types, such as head and neck cancer, prostate cancer, and gastric adenocarcinoma [13]. ICD is characterized by damage patterns, with docetaxel-treated xenograft tumors expressing MHC-I and calreticulin but lacking HMGB-1 or ATP, essential for immunogenic cell death [61]. The improved availability of cell surface antigens, such as the mannose-6-phosphate receptor, was able to increase granzyme B penetration into tumor cells treated with taxanes and T cell–targeted cell death in comparison to untreated tumor cells (Ramakrishnan *et al*.,) [62, 63]. Apart from increasing the susceptibility of tumor cells to T cell-mediated cell death, cetaxel has the potential to induce anticancer changes in the tumor immune microenvironment. Patients receiving paclitaxel as a neoadjuvant treatment for breast cancer had higher T cell numbers in posttreatment biopsies; a similar effect was also noted in tumors treated with docetaxel in mouse models [64, 65].

It has been demonstrated that taxanes' anticancer immunomodulatory effects enhance checkpoint inhibitor effectiveness in mice [66]. Drug exposure has an impact on taxanes' immunomodulatory characteristics as well. When paclitaxel and cisplatin were administered on a dose-dense, or metronomic, dosing schedule, intratumoral CD8+ T cell counts increased and therapeutic effectiveness improved [67].

It is possible to develop carefully engineered nanotherapeutics that allow for slow, sustained release of the same drug at the site of disease, which is comparable to the effects obtained with metronomic dosing, while also reducing systemic exposure to bioactive, unencapsulated drug [68-70].

In the case of docetaxel, which is a drug of choice for TNBC, Polysorbate 80 and ethanol use are linked to hypersensitivity events (*e.g.*, bronchospasm, rash, hypotension) and infusion-related toxicities. Patients are frequently premedicated with corticosteroids prior to docetaxel treatment to minimize the risk and severity of these reactions [14, 16].

## 8 NANOSOMAL DOCETAXEL LIPID SUSPENSION (NDLS)

NDLS represents a significant advancement in the treatment of TNBC. Recent clinical trials have demonstrated its enhanced efficacy and reduced toxicity compared to traditional Docetaxel formulations. NDLS’s ability to improve solubility and target tumor tissues more effectively makes it a promising candidate for further investigation. Moreover, its application in combination therapies offers new avenues to address drug resistance and heterogeneity in TNBC [15]

Current research on NDLS indicates potential benefits in improving Progression-Free Survival (PFS) and Overall Survival (OS) in TNBC patients. However, limitations such as production costs and scalability remain challenges. Comparing NDLS with other emerging therapies, such as immune checkpoint inhibitors and PARP inhibitors, highlights its unique role in enhancing drug delivery and reducing systemic exposure. Table **2** compares NDLS with Taxotere and Abraxane. This review aims to bridge existing research gaps and suggest future directions for NDLS development [71-77].

## 9 DOCETAXEL NANOSOMAL LIPID SUSPENSION: THERAPEUTIC POTENTIAL

The conventional treatment for primary operable breast cancer that is early/locally progressed is neoadjuvant chemotherapy, or NACT. Reaching pathological Complete Response (pCR) during Non-Acute Cardiotoxicity (NACT) has been linked to enhanced advantages for survival and is regarded as a proxy endpoint for survival results [78]. Despite improvements in cytotoxic chemotherapy for early-stage breast cancer, some patients continue to be at high risk for death and recurrence. Adjuvant chemotherapy, which is given in addition to the primary treatment, is often used in both neoadjuvant (before surgery) and adjuvant (after surgery) settings. Evidence indicates that whether chemotherapy is given before or after surgery, the survival outcomes are generally similar [79, 80]. The optimal treatment approach for stage III locally advanced breast cancer typically includes adjuvant chemotherapy, local radiation, and primary chemotherapy, with or without hormone therapy, followed by surgery if feasible. A phase II study is currently underway to evaluate the efficacy of administering four cycles of 100 mg/m^2^ docetaxel (Taxotere) *via* a one-hour intravenous infusion every three weeks. This treatment is followed by radiation, surgery, and four cycles of standard dose doxorubicin/cyclophosphamide (Cytoxan, Neosar) chemotherapy, with or without tamoxifen (Nolvadex). This paper presents preliminary results from 33 patients participating in the phase II trial [81].

With established efficacy and tolerability, docetaxel has become a preferred treatment for breast cancer. It is approved for use as an adjuvant therapy for operable node-positive breast cancer and for treating locally advanced or Metastatic Breast Cancer (MBC). Docetaxel has also proven to be a well-tolerated and effective neoadjuvant therapy for operable breast cancer. It has shown significant clinical activity as a single agent, achieving response rates of 34% and 50–72% for anthracycline-resistant and chemotherapy-naïve breast cancer, respectively. A phase III randomized trial for first-line metastatic breast cancer found that docetaxel (100 mg/m^2^) was more effective and better tolerated compared to doxorubicin (75 mg/m^2^). Current research is exploring the use of docetaxel alone or in combination with other drugs for early-stage or locally advanced breast cancer [82].

## 10 DOCETAXEL NANOSOMES LIPID SUSPENSION

**Fig. (2):**
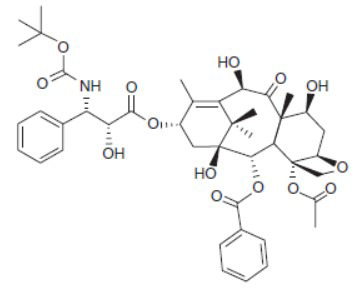
Molecular structure of docetaxel.

Over the past ten years, docetaxel has been identified as one of the most effective anticancer medications; nevertheless, its systemic toxicity and poor water solubility have severely restricted its clinical application. Recent advancements in nanotechnology have enabled the development of new drug delivery methods for docetaxel. These innovations can improve the drug's solubility in water, minimize adverse effects, and enhance targeted distribution to tumors through either passive or active targeting techniques [71]. It has been demonstrated that the second-generation taxoid cytotoxic drug Docetaxel (DTX) significantly inhibits the growth of numerous human malignancies. While working to improve the production of Taxol in the 1980s, Pierre Potier from the National Center for Scientific Research in France discovered Docetaxel (DTX). The process involved extracting the inactive precursor molecule 10-deacetylbaccatin-III from the needles of the European yew tree, *Taxus baccata*, a renewable resource. This precursor was used in the semisynthesis of DTX. Although DTX and paclitaxel share structural similarities, with the main differences being a hydroxyl group on carbon 10 and a tert-butyl carbamate ester on the phenylpropionate side chain (Fig. **2**), DTX has distinct chemical properties that result in greater water solubility compared to paclitaxel [83].

DTX stops physiological microtubule depolymerization and disintegration by attaching to and stabilizing tubulin, which leads to cell cycle arrest during the G2/M phase and eventual cell death. Additionally, DTX increases the expression of the cell cycle inhibitor p27 and inhibits the expression of the antiapoptotic gene Bcl2. These actions give DTX antitumor activity against a variety of cancers, including gastric, ovarian, breast, and prostate carcinomas, as well as non-small cell lung cancer [84-88]. Due to the excipients polysorbate 80 and ethanol in the conventional formulation of docetaxel, there are a number of toxicity concerns [14]. These include infusion-site reactions, acute cumulative fluid retention, severe anaphylactoid reactions, peripheral neuropathy, hypersensitivity reactions, and alcohol intoxication [89]. Although premedication with corticosteroids and antihistamines helps to overcome these toxicities, some patients may still have these side effects [90, 91]. To address concerns about toxicity, a new formulation of docetaxel, known as nanosomal docetaxel lipid suspension (NDLS, DoceAqualip, developed by Intas Pharmaceuticals Limited, India), was created. This formulation eliminates the use of polysorbate 80 and ethanol [15].

The FDA-recognized safe lipids (GRAS) were used in the development of the Nanosomal Docetaxel Lipid Suspension (NDLS) formulation, which completely omits ethanol and polysorbate 80. Compared to patients treated with Taxotere, patients treated with NDLS had higher systemic availability of docetaxel, according to a crossover study conducted on patients with solid tumors at a dose of 75 mg/m2. Furthermore, there was no discernible rise in toxicity when compared to Taxotere. The current efficacy research on breast cancer patients was conducted to evaluate the increased systemic availability of Nanosomal Docetaxel Lipid Suspension (NDLS). Preliminary results from this study were shared at the 2013 American Society of Clinical Oncology meeting [15].

Different methods are used to create drug delivery nanocarriers, which come in a variety of sizes, forms, materials, and configurations. Different nanocarriers have special qualities of their own that are crucial to the delivery of drugs in various ways [92]. Both active and passive methods can be used to deliver nanocarriers for DTX. The term “passive delivery” describes the movement of particles *via* convection and passive diffusion *via* leaky tumor capillary fenestrations into the interstitium and tumor cells [92]. It may be possible to create selective drug and particle accumulation by using the increased permeability and retention (EPR) effect. Fig. (**3**) highlights the passive targeting mechanism of NDLS. Active delivery, on the other hand, focuses on medication delivery to particular areas determined by molecular interactions. For example, a particle can be linked with a ligand to bind to its receptor, or it can be associated with an antibody to identify a particular antigen within the cells. Since tissues are continuously exposed to sublethal dosages of chemotherapy, a targeted drug delivery system may increase the anticancer efficacy of DTX, lessen its toxicity on normal cells, and delay the onset of Multidrug Resistance (MDR) [93].

Nanoparticles ranging between 100-150 nm exhibit optimal EPR-based tumor penetration. The median capillary pore size in solid tumors is ~500 nm, whereas CNS tumors exhibit a much smaller pore size (~12-14 nm). Alternative delivery strategies such as Convection-Enhanced Delivery (CED) and vascular normalization are under investigation in clinical trials to optimize drug transport and tumor penetration [53].

**Fig. (3):**
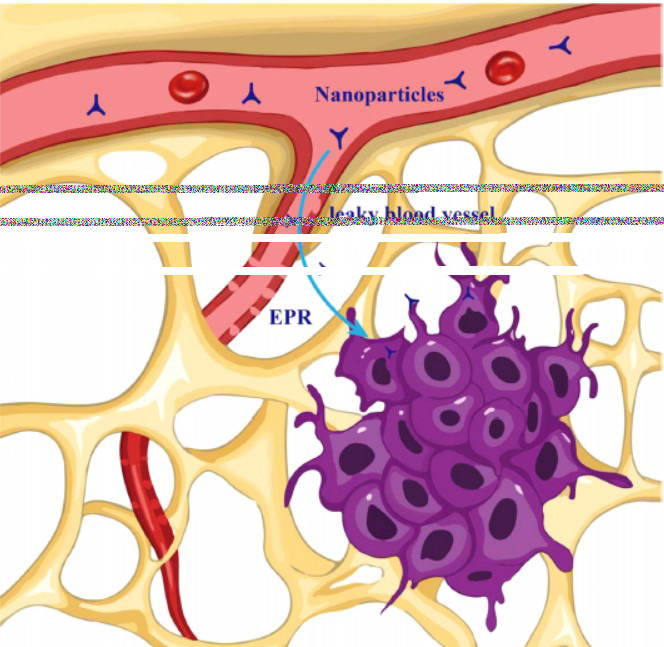
Passive targeting of nanoparticles for anti-tumor effects.

Drugs in lipid-based nanoformulations are either adsorbed onto the surface of nanoparticles or enclosed within a lipid core. These formulations, composed of biocompatible lipids and surfactants, include various types such as liposomes, Lipid-based Nanosuspensions (LNSs), Solid Lipid Nanoparticles (SLNs), and Nanostructured Lipid Carriers (NLCs) (Fig. **3**). Both liquid and solid biocompatible lipids can be sourced from natural or synthetic origins. Solid lipids include glycerides (*e.g.*, tripalmitin and glyceryl monostearate), fatty acids (*e.g.*, stearic acid), sterides (*e.g.*, cholesterol), and waxes (*e.g.*, microcrystalline wax). Common liquid lipids used are olive oil, soybean oil, oleic acid, and medium-chain triglyceride oil. Lipid-based nanoformulations are frequently used for delivering Docetaxel (DTX) because their biomimetic structures and lipid components enhance DTX's water solubility, improve drug membrane permeability, and enable sustained drug delivery [94, 95].

Liposomes are spherical vesicles composed of lipid bilayers surrounding an aqueous core, often used for encapsulating hydrophobic, amphiphilic, or water-soluble drugs. There are several methods for creating liposomes, including aqueous solution techniques, pH gradient methods, ammonium sulfate gradients, and the use of organic solvents. PEGylated liposomes were introduced to enhance circulation time and reduce clearance after intravenous administration. While trials are ongoing for liposome-encapsulated Docetaxel (DTX), challenges such as limited storage stability, rapid drug release, and low encapsulation efficiency remain barriers to its broader application [96].

A new type of lipid-based nanocarrier, lipid-based Nanosuspensions (LNS), was introduced, using DTX as a model drug. LNS production without organic solvents allows efficient large-scale manufacturing and therapeutic use [97, 98]. Our lab developed LNSs, colloidal dispersions of nanoscale drug particles in aqueous media. Surfactants from lipids stabilize LNSs, preventing self-aggregation. LNSs offer benefits like large-scale production, high drug-loading capacity, long-term stability, low toxicity, and compatibility with water and oil-soluble medications, making them a promising nanoformulation [99]. Manufacturing methods for these formulations include high-pressure homogenization, precipitation, media milling, emulsion/microemulsion templating, and dry co-grinding [100].

DTX delivery techniques like SLN and DTX-loaded PEGylated-NPs show satisfactory anticancer effects in colon and liver cancer cases due to enhanced bioavailability, saturation solubility, and dissolving rate of weakly soluble medications [101]. Nevertheless, there haven't been many reports on the antitumor efficacy assessment of DTX-loaded nanosuspensions against malignant melanoma. DTX-loaded LNS was initially created in our studies to treat malignant melanoma in mice.

To prepare the LNS, high-pressure homogenization was employed. Particle size, morphology, and zeta potential were all described. Dialysis bag diffusion was used to measure *in vitro* medication release. Duopafei® and LNS were tested for their *in vitro* cytotoxicity on SKOV-3 and B16 cells. Lastly, Kunming mice expressing B16 cells were used to assess the pharmacokinetics, tissue distribution, and *in vivo* anticancer activity of the medication. For DTX delivery, high-pressure homogenization was used to create a folate-targeted docetaxel-lipid-based nanosuspension (tLNS), a poly(ethylene glycol)-modified docetaxel-lipid-based nanosuspension (pLNS), and a DTX-loaded LNS (DTX-LNS) [99].

## 11 CLINICAL STUDIES OF NANOSOMAL LIPID SUSPENSION: DOCETAXEL

Nanosomal Docetaxel Lipid Suspension (NDLS) consists of uniformly sized docetaxel microparticles dispersed in a lipid-based formulation. This lipid-based formulation improves the safety profile of docetaxel by eliminating ethanol and polysorbate 80, which are present in traditional formulations such as Taxotere®. A randomized, open-label study (NCT03671044) [96] is underway to assess the effectiveness and safety of NDLS at doses of 75 mg/m^2^ and 100 mg/m^2^ compared to Taxotere® at 100 mg/m^2^ in patients with triple-negative breast cancer who have locally advanced or metastatic disease. Patients will continue treatment if the disease remains stable and toxicity levels are manageable. Study results have not yet been reported.

In another study [73], the effects of conventional docetaxel-based chemotherapy and Nanosomal Docetaxel Lipid Suspension (NDLS)-based chemotherapy were compared in patients with primary operable breast cancer. Between September 2020 and August 2021, Omega Hospitals in Hyderabad, Telangana, India, served as the site of this prospective observational study. Patients aged 18 years and older with newly diagnosed, histologically confirmed stage IIb or IIIa breast cancer, regardless of subtype (hormone receptor [HR]-positive or -negative, human epidermal growth factor receptor 2 [HER^2^]-positive or -negative, or triple-negative breast cancer [TNBC]), and an Eastern Cooperative Oncology Group (ECOG) performance status of 0–2 were included in the study.

Individuals with multiple cancers or those who were pregnant were excluded. Patients were randomly assigned to receive either NDLS or conventional docetaxel, based on the oncologist's discretion. The study indicated that NDLS-based regimens offered improved safety profiles and similar response rates compared to conventional docetaxel. It was suggested that NDLS might have a better tolerability profile at equivalent doses due to an Enhanced Permeability and Retention (EPR) effect, potentially allowing more effective drug delivery to tumors without requiring corticosteroid premedication.

This study was a parallel, open-label, randomized, multiple-dose trial designed for patients with metastatic or locally advanced breast cancer who had experienced treatment failure. The study included women aged 18 to 65 with histopathologically or cytologically confirmed breast cancer that had progressed locally or distantly after previous chemotherapy. These patients met the Response Evaluation Criteria in Solid Tumors (RECIST) 1.1, had at least one measurable lesion, demonstrated adequate bone marrow, renal, and hepatic function, and had a life expectancy of at least six months. Patients with an Eastern Cooperative Oncology Group (ECOG) performance status of 2 were included. They were randomized in a 2:1 ratio to receive either Taxotere or NDLS. The trial enrolled a total of 72 patients. The overall therapeutic response rate (complete + partial) was 35.5% for Taxotere and 26.3% for NDLS, suggesting a potentially stronger response with Taxotere. Despite the lack of premedication in the NDLS group, safety outcomes were comparable to those observed with Taxotere [15].

## 12 NANOSOMAL DOCETAXEL LIPID SUSPENSION IN OTHER TYPES OF CANCERS

A previous case report [102] described a pregnant woman with metastatic breast cancer that had spread to her liver and lungs.

For breast cancer in pregnant women, chemotherapy regimens based on anthracyclines and taxanes are typically used. Dacateaxel has demonstrated a favorable toxicity profile during the second and third trimesters of pregnancy. Since 2013, India has approved a novel Nanosomal Docetaxel Lipid Suspension (NDLS) named DoceAqualip for treating advanced solid tumors due to its proven efficacy and safety. In this case, the pregnant patient underwent six cycles of an NDLS-based TAC regimen and exhibited signs of partial response. At 32 weeks of gestation, she delivered a healthy baby boy with normal weight and Apgar scores.

In metastatic Castration-Resistant Prostate Cancer (mCRPC), a standard regimen is docetaxel at 75 mg/m^2^ every 3 weeks. This regimen has been observed after 20 cycles of biweekly Nanosomal Docetaxel Lipid Suspension (NDLS) in patients with mCRPC [103].

Three cases of long-term NDLS treatment (>20 cycles) from Jawaharlal Nehru Cancer Hospital & Research Centre, Bhopal, India, are discussed. The patients received biweekly NDLS at 45 mg/m^2^ for 22, 36, and 40 cycles, except one patient who started at 50 mg/m^2^ before dose reduction. All showed a >50% decline in PSA levels, with a time to Treatment Failure (TTF) of 14.8, 18.2, and 20.6 months, respectively. The Overall Survival (OS) was 21.6, 22.2, and 25.8 months. Common side effects included anemia, lymphopenia, and neutropenia. NDLS was well-tolerated without new safety concerns. Biweekly NDLS for over 20 cycles was effective and well-tolerated in mCRPC patients, suggesting its potential for long-term management.

A multicenter, retrospective study evaluated the efficacy and safety of Nanosomal Docetaxel Lipid Suspension (NDLS) for treating metastatic epithelial ovarian carcinoma. The analysis reviewed records of patients who received NDLS (60-75 mg/m^2^ every three weeks) between September 2014 and September 2018. The study assessed the Overall Response Rate (ORR), disease control rate based on RECIST 1.1 criteria, Overall Survival (OS), and safety. Among the 13 patients reviewed, 46.2% received NDLS as first-line therapy, and 53.8% as second-line therapy (57.1% were platinum-sensitive, and 42.9% were platinum-resistant). The ORRs were 60.0% for first-line and 57.1% for second-line treatments. Median OS was 17.4 months for first-line, 26.1 months for platinum-sensitive, and 14.8 months for platinum-resistant patients. The study reported no grade III/IV adverse events, with grade I-II events occurring in 84.6% of patients. Overall, NDLS-based chemotherapy was effective and well-tolerated for managing metastatic epithelial ovarian carcinoma [101].

In this retrospective, multicenter study across six centers, the medical records of adult patients treated with NDLS (75 mg/m^2^ every 3 weeks) for sarcoma were reviewed [104]. The efficacy outcomes assessed were the Overall Response Rate (ORR), which includes both complete and partial responses, and the Disease Control Rate (DCR), which encompasses complete and partial responses plus stable disease, across neoadjuvant and metastatic settings. Overall Survival (OS) and safety were also analyzed. Out of 11 patients (1 neoadjuvant, 3 adjuvant, 7 metastatic), the majority had leiomyosarcoma (63.6%), with the remainder having various other sarcoma types. NDLS was administered with gemcitabine to 10 patients and with cyclophosphamide to 1 patient. Efficacy was assessed in 7 patients (1 neoadjuvant, 6 metastatic). In the neoadjuvant setting, one patient achieved a complete response. For metastatic cases, the ORR was 50% and the DCR was 66.7%. The median follow-up period was 6.5 months, and the median OS in the metastatic setting was 15.8 months. Common side effects included neutropenia, thrombocytopenia, lymphopenia, anemia, nausea, vomiting, and diarrhea. NDLS was well tolerated with no new safety issues reported. NDLS-based chemotherapy proved effective and well-tolerated for sarcoma treatment, but additional prospective studies are needed to validate these results.

The most prevalent cancer is Squamous Cell Carcinoma of the Head and Neck (SCCHN) among Indian men. Docetaxel, used alone or in combination with other drugs, is a standard treatment for Squamous Cell Carcinoma of the Head and Neck (SCCHN). This multicenter, retrospective study evaluated the efficacy and safety of a new formulation, Nanosomal Docetaxel Lipid Suspension (NDLS), in treating SCCHN. The study reviewed medical records of patients who underwent NDLS-based chemotherapy from August 2014 to September 2018. Efficacy data were available for 30 of the 34 patients (20 of 23 for induction therapy and 10 of 11 for palliative therapy). NDLS-based induction chemotherapy demonstrated a notable 95% Overall Response Rate (ORR) and Disease Control Rate (DCR), with a median Overall Survival (OS) of 43.5 months. In the palliative setting, the ORR and DCR were 50%, with a median OS of 4.6 months. Adverse events were observed in 82.6% of patients, but no new safety issues were identified. Overall, NDLS-based chemotherapy was effective and well-tolerated for SCCHN treatment [15].

The study titled “Efficacy and Safety of Nanosomal Docetaxel Lipid Suspension-Based Chemotherapy in Gastric and Gastroesophageal Junction Adenocarcinoma” [96] evaluated the effectiveness and safety of nanosomal docetaxel lipid suspension (NDLS; DoceAqualip) in treating patients with gastric and Gastroesophageal Junction (GEJ) adenocarcinoma. The study included 43 patients, with results showing that NDLS-based chemotherapy had a promising Overall Response Rate (ORR) of 58.82% and a Disease Control Rate (DCR) of 94.11% in the neoadjuvant setting. For metastatic cases, the ORR was 77.77% and the DCR was 83.33%. The median Overall Survival (OS) was 31.9 months for metastatic patients, though no median OS was reached for the neoadjuvant group. NDLS was generally well-tolerated, with common side effects including anemia, lymphopenia, and nausea, but no new safety issues were identified. Overall, NDLS-based chemotherapy was found to be effective and well-tolerated for gastric and GEJ adenocarcinoma.

A case report by Naik *et al*. [105] contributes to the evidence that standard docetaxel formulations containing polysorbate 80 and ethanol can cause adverse effects, even when corticosteroids and antihistamines are administered beforehand. In contrast, NDLS (Doceaqualip) may offer a valuable alternative for treating prostate cancer that has metastasized to the bone. Thus, DoceAqualip has been shown to be safe and effective in treating various cancer types. The authors describe a patient with Stage IIIB cervical cancer who achieved a complete response to concurrent Chemoradiotherapy (ChemoRT) with carboplatin and DoceAqualip, without experiencing any severe side effects [106].

## CONCLUSION

Triple-Negative Breast Cancer (TNBC) is one of the most aggressive subtypes of breast cancer, characterized by high recurrence rates and a lack of targeted therapies. Conventional chemotherapy, particularly docetaxel, has been a cornerstone of TNBC treatment. However, its effectiveness is often compromised due to systemic toxicity, variable patient response, and emerging drug resistance.

Nanosomal Docetaxel Lipid Suspension (NDLS) offers a promising alternative by utilizing lipid-based nanotechnology to enhance drug delivery, improve tumor targeting, and minimize adverse effects. Unlike conventional docetaxel formulations, NDLS eliminates the need for ethanol and polysorbate 80, reducing hypersensitivity reactions and improving patient tolerability. Its optimized pharmacokinetics allow for sustained drug release and deeper tumor penetration, potentially overcoming chemotherapy resistance mechanisms.

To establish NDLS as a standard treatment option, large-scale clinical trials are necessary to confirm its long-term benefits and safety. Additionally, exploring its combination with targeted therapies, immunotherapies, and anti-angiogenic agents may further enhance treatment efficacy. Expanding its application beyond TNBC to other aggressive cancers could also provide new therapeutic opportunities.

This review highlights NDLS as a significant advancement in TNBC therapy, paving the way for future research and clinical integration. By improving drug efficacy while reducing toxicity, NDLS has the potential to transform chemotherapy strategies, ultimately leading to better patient outcomes and an improved quality of life for those battling TNBC.

## AUTHORS' CONTRIBUTIONS

The authors confirm their contributions to the paper as follows: PJ and RC conceived and designed the study; IP was responsible for data curation; VA and AK performed the analysis and interpretation of results; FA handled writing, reviewing, and editing; and PJ prepared the original draft. All authors reviewed the results and approved the final version of the manuscript.

## Figures and Tables

**Fig. (1):**
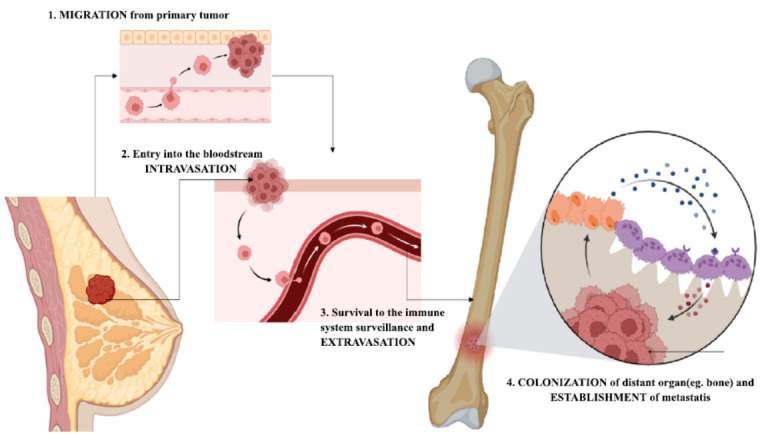
Cinematic view of one metastasis from breast cancer. This figure illustrates a basal-like subtype of TNBC, characterized by its high mitotic index and invasive morphology. Gross morphology includes irregular tumor borders and dense cellular proliferation.

**Table 1:** FDA-approved epigenetic modulators (Epidrugs) and their targets in cancer therapy.

**ClinicalTrials.govID**	**Condition**	**Drug /Formulations**	**Study Design**	**Brief ** **Description**	**Phase of Trial**	**References**
NCT01928576	Non-Small Cell Lung Cancer Epigenetic Therapy	Drug: Azacitidine Drug: Entinostat Drug: Nivolumab Drug: CC-486 300	Allocation: Randomized Interventional Model: Parallel Assignment Masking: None (Open Label) Primary Purpose: Treatment	Response Rate	Phase 2	[31]
NCT03179943	Urothelial Carcinoma	Drug: Atezolizumab Drug: Guadecitabine	Primary Purpose: Treatment Allocation: N/A Interventional Model: Single Group Assignment Masking: None (Open Label)	The Phase II research aims to determine the optimal dose of guadecitabine and atezolizumab combination therapy for patients with stage IV recurrent or advanced urothelial carcinoma. The study will enroll 4-5 patients, based on safety run-in and subject replacement.	Phase 2	[32]
NCT03164057	Acute Myeloid Leukemia Myelodysplastic Syndromes	Drug: Azacitidine Drug: Decitabine Drug: Cytarabine	Primary Purpose: Treatment Allocation: Randomized Interventional Model: Parallel Assignment Masking: None (Open Label)	In order to gather preliminary biology and clinical data, this study intends to evaluate the safety and therapeutic effectiveness of epigenetic priming with DNA methyltransferase inhibitors prior to chemotherapy blocks. It will also evaluate the overall reduction in DNA methylation and result.	Phae 2	[33]
NCT04190056	Anatomic Stage IV Breast Cancer AJCC v8 Prognostic Stage IV Breast Cancer AJCC v8	Biological: Pembrolizumab Drug: Tamoxifen Drug: Vorinostat	Allocation Randomized Interventional Model Parallel Assignment Masking None (Open Label) Primary Purpose Treatment	This phase II trial investigates the effectiveness of pembrolizumab and tamoxifen in treating estrogen receptor-positive breast cancer. Pembrolizumab boosts immune defenses, while tamoxifen prevents tumor cell growth. Vorinostat inhibits cell growth enzymes, aiming to find a combination that reduces dosage while improving estrogen receptor-positive breast cancer control.	Phase 2	[34]
NCT00828854	ER + Breast Cancer	Drug: Entinostat Drug: Aromatase Inhibitor (AI) Therapy	**Primary Purpose: **Treatment **Allocation: **N/A **Interventional Model: **Single Group Assignment **Masking: **None (Open Label)	By drastically lowering estrogen receptor-α activity and aromatase inhibitor resistance, entinostat may be added to AI, potentially raising the therapeutic benefit rate from 5% to 25% while maintaining a tolerable safety profile.	Phase 2	[35]
NCT00676663	Breast Cancer Estrogen Receptor-Positive Breast Cancer Breast Cancer, Estrogen Receptor-Positive ER+ Breast Cancer	Drug: entinostat Drug: exemestane Drug: Placebo	Double blind	This study aims to assess the safety and effectiveness of exemestane plus entinostat in the treatment of advanced breast cancer.	Phase 2	[36]
NCT03671044	TNBC	Drug: Nanosomal Docetaxel Lipid Suspension Drug: Nanosomal Docetaxel Lipid Suspension Drug: Taxotere®	Randomized, open-label	NDLS *vs*. conventional docetaxel in metastatic TNBC	Phase 3	[37]

**Table 2:** Comparison of key properties among Nanosomal Docetaxel Lipid Suspension (NDLS), Taxotere (Conventional Docetaxel), and Abraxane (Paclitaxel Nanoparticles).

**Property**	**NDLS (Nanosomal Docetaxel Lipid Suspension) [72, 73]**	**Taxotere (Conventional Docetaxel) [74, 75]**	**Abraxane (Paclitaxel Nanoparticles) [74, 76, 77]**
Formulation Type	Lipid-based nanosuspension	Polysorbate 80 + Ethanol	Albumin-bound nanoparticles
Particle Size	Approximately 100 nm	Non-nano	Approximately 130 nm
Zeta Potential	Approximately -30 mV	Not applicable	Approximately -10 mV
Solvent Requirement	None	Requires ethanol-based solvent	None
Toxicity Profile	Reduced hypersensitivity reactions	Higher risk of hypersensitivity	Lower neurotoxicity
Need for Corticosteroid Premedication	No	Yes	No

## LIST OF ABBREVIATIONS

BLIA: Basal-Like Immune-Activated
CPGs: Cancer Predisposition Genes
DCR: Disease Control Rate
DFS: Disease-Free Survival
DNMTs: DNA Methyltransferases
FDA: Food and Drug Administration
HDACis: Histone Deacetylases
LAR: Luminal Androgen Receptor
LNSs: Lipid-based Nanosuspensions
MBC: Metastatic Breast Cancer
MSL: Mesenchymal Stem-Like
NACT: Non-Acute Cardiotoxicity
NDLS: Nanosomal Docetaxel Lipid Suspension
NLCs: Nanostructured Lipid Carriers
SLNs: Solid Lipid Nanoparticles
TME: Tumor Microenvironment
TNBC: Triple-Negative Breast Cancer
VEGF: Vascular Endothelial Growth Factor
